# Microfluidic and impedance analysis of rosemary essential oil: implications for dental health

**DOI:** 10.1186/s12938-024-01301-4

**Published:** 2024-11-04

**Authors:** Karunan Joseph, Bojan Petrović, Syarifah Aisyah Syed Ibrahim, Aung Thiha, Lazar Milić, Mohd Yazed Ahmad, Nebojša Pavlović, Sanja Kojić, Fatimah Ibrahim, Goran M. Stojanović

**Affiliations:** 1 Centre for Innovation in Medical Engineering (CIME), Universiti Malaya, 50603 Kuala Lumpur, Malaysia; 2 Department of Dental Medicine, Faculty of Medicine, University of Novi Sad, Novi Sad, Serbia; 3 Faculty of Technical Science, University of Novi Sad, Novi Sad, Serbia; 4 Department of Pharmacy, Faculty of Medicine, University of Novi Sad, Novi Sad, Serbia

**Keywords:** Rosemary essential oil, Microfluidics, Electrochemical impedance spectroscopy, Dental health, Antimicrobial properties, Oral care

## Abstract

**Background:**

Oral health is closely linked to systemic conditions, particularly non-communicable diseases (NCDs), which can exacerbate oral issues. Essential oils (EOs) have emerged as potential alternatives for oral health due to their antibacterial, anti-inflammatory, and antioxidant properties. Among these, rosemary essential oil (REO) shows promise due to its various biological activities. This study investigates the potential of REO in dental applications using microfluidic devices and electrochemical impedance spectroscopy (EIS) to analyze the electrical properties of REO in artificial saliva (AS) mixtures.

**Results:**

The study demonstrated significant variations in impedance across different REO concentrations and their mixtures with AS. Higher impedance was observed in REO mixtures, particularly at lower frequencies, indicating distinct electrical properties compared to pure AS. The impedance of REO was influenced by its concentration, with a 1% REO solution showing higher impedance than a 4% solution, possibly due to micelle formation and changes in dielectric properties. Additionally, microfluidic devices enabled precise control over fluid interactions and real-time monitoring, offering valuable insights into REO's behavior in a simulated oral environment. The impedance data demonstrated significant differences in REO–AS mixtures, highlighting potential interactions critical for oral care applications.

**Conclusions:**

Rosemary essential oil exhibits unique electrical properties, making it a promising candidate for dental applications, particularly in preventing and treating oral diseases. Microfluidic devices enhance the accuracy and reliability of studying REO's interactions with AS, providing a robust platform for future dental research. The findings suggest that REO could be effectively incorporated into oral care products, offering a natural alternative for combating oral pathogens, reducing inflammation, and protecting against oxidative stress. Future research should focus on clinical trials to validate these findings and explore the synergistic effects of REO with other essential oils.

**Supplementary Information:**

The online version contains supplementary material available at 10.1186/s12938-024-01301-4.

## Background

Oral health problems associated with systemic conditions include a higher prevalence of oral diseases in individuals with limited ability for oral self-care, such as those with non-communicable diseases (NCDs) [1]. The association between oral and systemic health is bidirectional, with evidence supporting a greater prevalence of oral conditions in people suffering from NCDs [2]. Some of the main systemic conditions associated with oral health problems include five types of cancer: diabetes mellitus, cardiovascular diseases, depression, neurodegenerative conditions, rheumatic diseases, inflammatory bowel disease, gastric helicobacter pylori, obesity, and asthma. Other systemic diseases affecting oral health include heart disorders, hypertension, blood disorders, stroke, kidney disorders, thyroid disorders, liver disorders, and others [3]. These systemic diseases can manifest in the oral cavity and harm individuals' overall well-being and quality of life [4]. Treatment of systemic diseases, such as cancer treatment and certain medications, can also cause oral lesions.

Oral hygiene is not just a routine; it is a crucial aspect of an individual's quality of life and overall health. Maintaining good oral health through proper oral hygiene practices, such as regular brushing and dental visits, is the first defense against various oral diseases. It can prevent discomfort, pain, tooth loss, impaired oral functioning, and disfigurement, significantly impacting an individual's quality of life. Additionally, oral health is closely linked to social relationships and self-esteem. A healthy mouth and teeth contribute to positive social interactions and improved self-esteem. In contrast, oral health issues like missing teeth or bad breath can cause embarrassment and affect an individual's confidence. Therefore, maintaining good oral hygiene is not just about preventing diseases but promoting overall health and enhancing an individual's quality of life [5, 6].

Essential oils (EOs) affect oral health through various mechanisms. EOs possess antibacterial properties, inhibiting the growth of common oral bacteria and reducing the formation of oral plaque [7–9]. They also have anti-inflammatory effects, modulating the immune response and reducing inflammation associated with periodontitis [10]. EOs can permeabilize bacterial membranes and inhibit the activity of efflux pumps, contributing to their antibacterial activity [11]. Additionally, EOs can downregulate gene expression in the inflammatory response. Some specific compounds found in EOs, such as carvacrol, γ-terpinene, and *p*-cymene, have been identified as potential targets for virulence proteins of *Streptococcus mutans*, a bacteria associated with dental caries. Overall, EOs offer a promising alternative therapy for common oral diseases, but further research is needed to determine their full potential and safety.

Clove oil, cinnamon oil, turmeric oil, nutmeg oil, and peppermint oil effectively combat oral pathogens [12]. Clove oil was the most effective against *Streptococcus mutans*, *Candida albicans*, and *Enterococcus faecalis* [13]. Turmeric oil and peppermint oil were also effective against *Streptococcus mutans* [14]. Cinnamon oil was effective against *Candida albicans* and *Enterococcus faecalis* [15]. Lime, lemon, and orange essential oils, particularly C. sinensis, showed antimicrobial activity against Candida spp. and weak antibacterial activity against various oral pathogens [16]. *Thymus fallax* essential oil demonstrated significant antimicrobial and anti-biofilm efficacy against common oral pathogens. A combination of essential oils, including virgin coconut oil, eucalyptus oil, peppermint oil, thyme oil, and clove oil, showed potent antibacterial activity against Porphyromonas gingivalis. Various essential oils exhibited antimicrobial activity against opportunistic pathogenic bacteria and *Candida albicans*.

Rosemary essential oil (REO) has potential therapeutic applications due to its various biological activities. It has been found to have antioxidant activity closely related to its verbenone content [17]. REO also exhibits anti-proliferative activity against certain cancer cell lines, such as human pancreatic cancer cell line SW1990 and gastric epithelial cell line NCI-N87. Additionally, rosemary extract, which contains carnosic acid, is a potent opener of heteromeric KCNQ3/5 channels, suggesting unique therapeutic potential for neurological disorders [18]. Rosemary has also been found to have antitumor, antibacterial, antiviral, antifungal, antiprotozoal, anti-inflammatory, antioxidant, immunomodulatory, and analgesic properties [19]. Furthermore, rosemary essential oil has been shown to alleviate liver toxicity and testis damage induced by certain compounds [20]. Encapsulation of rosemary essential oil in zein nanoparticles has been explored as a potential nano-drug delivery system to enhance its stability and bioavailability. These characteristics make rosemary oil an intriguing candidate for oral health applications, potentially aiding in preventing and managing oral diseases. Moreover, the pleasant aroma of rosemary oil could enhance user acceptance of oral care products.

Rosemary essential oil has several properties, making it a potential treatment for dental conditions. It has antimicrobial effects, which can help combat pathogens such as *Streptococcus mutans*, a major cause of caries. The essential oil also has antioxidant activity, which can scavenge reactive oxygen species and protect against oxidative damage [17]. Additionally, rosemary essential oil has anti-inflammatory effects, which can help reduce inflammation in the oral cavity [21]. It also exhibits analgesic effects, providing pain relief [22]. Furthermore, the essential oil has been found to have anti-proliferative activity against cancer cell lines, suggesting its potential in preventing oral cancer [23]. These properties make rosemary essential oil a promising candidate for dental treatments, including the prevention and treatment of caries, inflammation, and oral cancer.

Microfluidic devices can be used to improve the efficiency of dental research in several ways. Firstly, they enable the integration of complex systems in a miniaturized state, allowing for the manipulation of fluids at a cellular scale and the control of flow rates [24]. This level of precision is particularly advantageous and beneficial in pharmaceutical research, as it aids in developing drugs, optimizing dosages, and creating controlled release systems. This provides a dynamic environment for conducting experiments and monitoring analytes. Secondly, microfluidic devices can be used for point-of-care diagnostic systems, particularly using saliva as a diagnostic fluid [25]. Saliva testing is easy and accessible, and microfluidics-based methods can save time and cost compared to traditional methods. Microfluidic devices greatly facilitate real-time monitoring and analysis, allowing researchers to observe and analyze dynamic processes unfolding in real time. Thirdly, microfluidic devices can study dental biofilm formation, offering control over multiple microenvironmental factors and allowing for quantitative analysis of bacteria growth and biofilm development [26]. This provides insights into the conditions promoting colonization and biofilm formation, vital for understanding tooth decay and related health problems. Overall, microfluidic devices offer a versatile and efficient platform for dental research. Lastly, microfluidic systems can perfectly replicate and simulate physiological conditions, allowing researchers to study complex interactions in a highly controlled environment. In dental research, this is essential as it aids in understanding the dynamics of oral processes and allows for testing the efficacy of oral care products under realistic and true-to-life conditions [27, 28].

The research aims to explore the mixing properties and electrical characteristics of rosemary essential oil (REO) in combination with artificial saliva. This involves investigating their interaction within microfluidic systems and utilizing electrochemical impedance spectroscopy (EIS) to analyze the electrical behavior of the mixtures. Changes in impedance (resistance) will be examined to gain insights into the electrochemical interactions, focusing on correlating these electrical properties with the composition of the REO–saliva mixtures. The findings may have implications for the solution's therapeutic potential.

## Results

This section presents the impedance analysis conducted using two devices—SmartMF and DropSens—across multiple liquids and their mixtures, utilizing various electrodes. The findings are categorized by measurement type (outside disk and on disk), instrument, and the electrode used (Table 1), providing a comprehensive view of the impedance characteristics across different conditions.

**Table 1:** Clarification of measurements over electrode type and instrument used

Electrode	DropSens	SmartMF
CIME electrode	X	X
Carbon electrode	X	–
Interdigital Al electrode	–	X

### DropSens measurements on CIME electrode

**Fig. 1:**
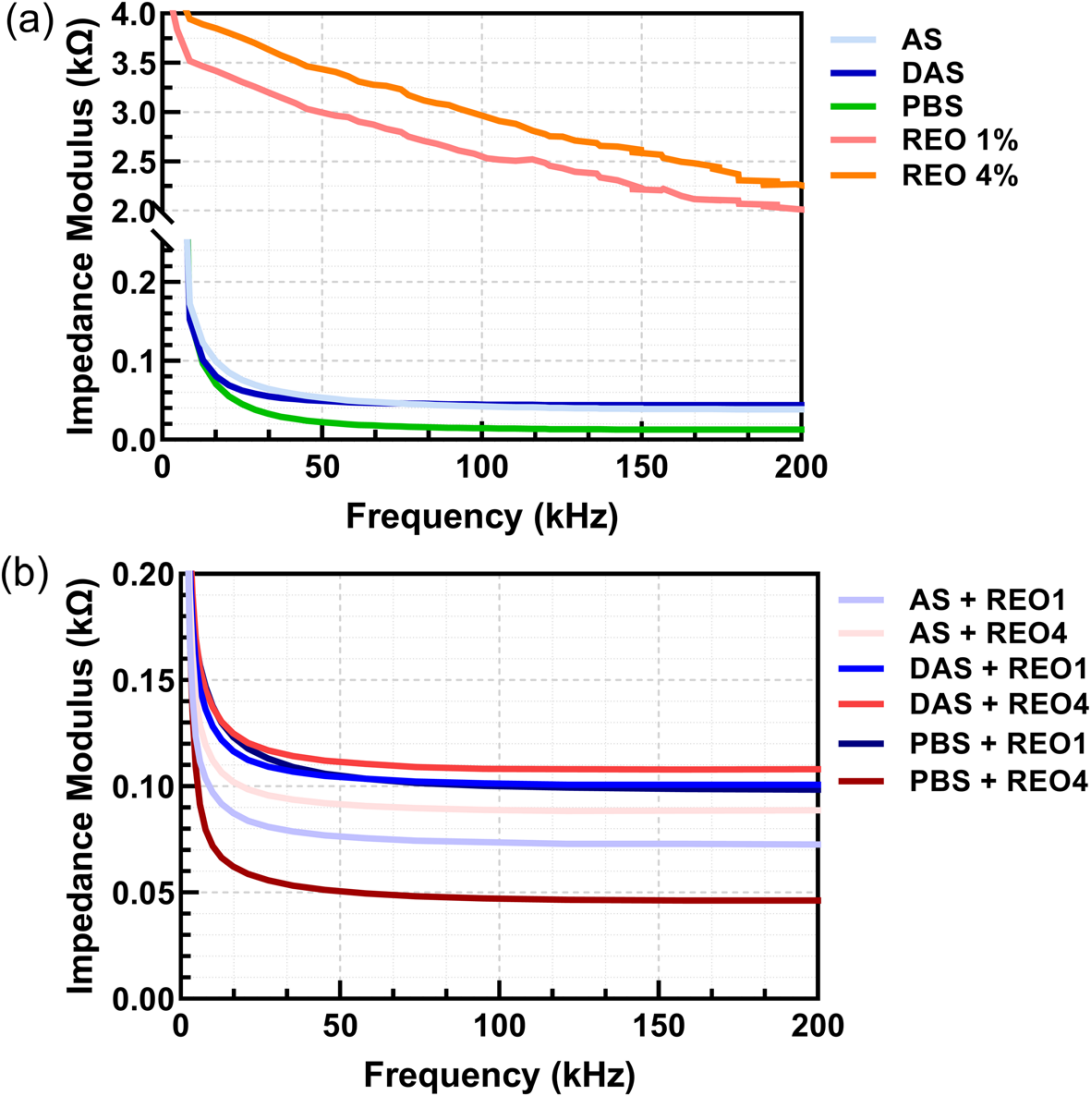
Graphs depicting impedance values in Ohms across the frequency range between 500 and 200,000 Hz for the empty electrode in **a** 5 liquids and **b** their mixtures with REO1 and REO4. Out of the disk, DropSens measurements

We observed significant fluctuations (Fig. 1) in the impedance values; notably, the REO1 and REO4 presented the highest average impedance, while PBS showed the minimal mean impedance. In the middle frequency, the impedance measurements showed significant variation, with the empty electrode recording the highest mean impedance and PBS the lowest. This variability trend continued into higher frequencies, where the empty electrode had the highest average impedance and REO4 the lowest. The reduced standard deviations in these higher frequencies indicate a more consistent impedance across samples. Pairwise *t*-tests across all frequency bands revealed significant differences in impedance values, underscoring the distinct electrical properties of each liquid and mixture. Notably, REO4 demonstrated the most significant variability, particularly at lower frequencies, likely due to its unique composition and interactions. Additionally, mixtures like PBS REO1 and AS REO4 showed significantly different impedance properties from their single components, highlighting the influence of mixing on electrical characteristics—essential for applications that require specific impedance behaviors.

### SmartMF measurements on CIME electrode

**Fig. 2:**
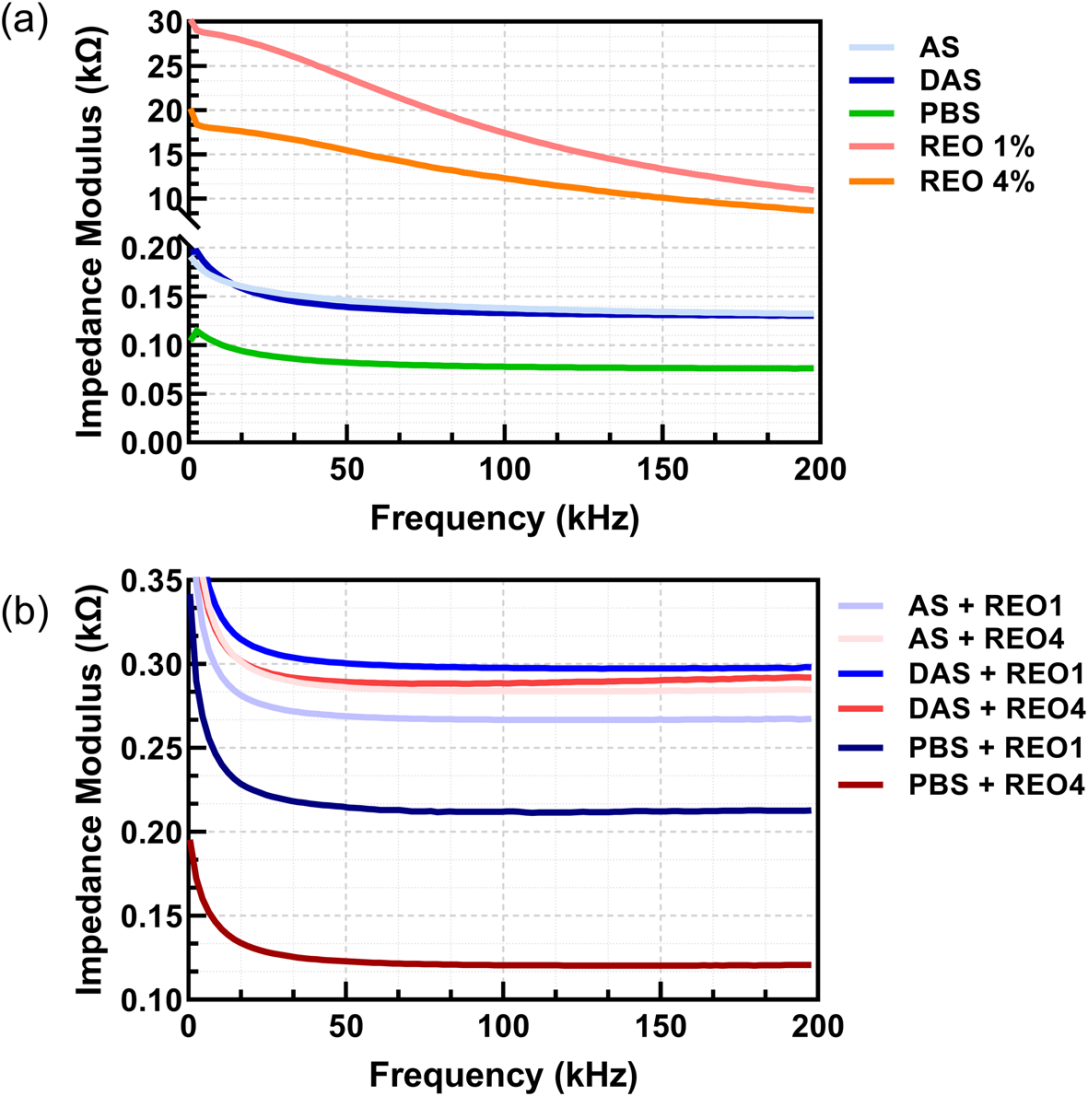
Graphs depicting impedance values in Ohms across the frequency range between 500 and 200,000 Hz for the empty electrode: **a** 5 liquids; **b** their mixtures with REO1 and REO4

The impedance measurements on the CIME electrode were categorized into three frequency ranges: lower frequencies (500 to 20,000 Hz), middle frequencies (20,000 to 100,000 Hz), and higher frequencies (100,000 to 200,000 Hz). For the empty electrode, the mean impedance values were notably high across all frequency ranges. In comparison, all solutions and mixtures exhibited lower impedance values. All graphs representing impedance values are shown in Fig. 2. REO1 and REO4 showed higher impedances than the other solutions.

When mixed with PBS, REO1 and REO4 mixtures exhibited decreased impedance values. The mixtures of AS and DAS with REO1 and REO4 also showed reduced impedance values. The DAS REO4 ranged from 334.01 ± 29.88 Ω at lower frequencies to 299.33 ± 20.78 Ω at higher frequencies, indicating consistent impedance reductions across frequencies for all mixtures (Table 2).

**Table 2:** Impedance measurement summary SmartMF

Sample	Lower frequency mean	Lower frequency SD	Middle frequency Mean	Middle frequency SD	Higher frequency Mean	Higher frequency SD
Empty electrode	823,647.50	4124.95	113,660.81	154.43	35,074.95	55.10
PBS	103.99	7.62	83.91	5.80	78.52	5.50
AS	184.15	2.89	158.76	4.71	147.29	7.24
DAS	182.62	4.29	147.07	2.81	138.18	1.76
REO1	27,056.34	2383.69	21,623.89	1406.73	13,379.15	457.81
REO4	14,363.74	6648.40	12,243.78	4619.38	8944.94	2481.19
PBS REO1	216.12	10.66	184.11	7.81	180.63	7.36
PBS REO4	154.99	5.73	129.93	4.23	127.00	3.84
AS REO1	314.87	10.82	280.94	9.00	277.99	8.75
AS REO4	337.99	9.60	296.13	7.11	292.62	6.47
DAS REO1	347.43	6.06	308.66	4.08	304.89	3.88
DAS REO4	334.01	29.88	299.36	24.27	299.33	20.78

The intergroup comparison of impedance values across 12 samples, including the empty electrode, various liquids (PBS, AS, DAS, REO1, REO4), and their mixtures, revealed several statistically significant differences. The results of pairwise *t*-tests for the lower, middle, and higher frequency ranges indicate that most comparisons exhibit significant differences (*p* < 0.001) across the samples. This suggests that each liquid and its mixture exhibit distinct impedance characteristics compared to others. Notably, the empty electrode consistently showed significant differences in impedance values compared to all other samples, highlighting its distinct baseline properties.

Furthermore, specific pairings such as REO1 and REO4 and their mixtures with PBS, AS, and DAS also demonstrated significant differences across most frequency ranges. This implies that the composition of these liquids and their interactions in mixtures substantially influence the impedance measurements. The consistent significant differences in many pairwise comparisons suggest that the impedance measurement technique is sensitive to the unique electrical properties of each liquid and mixture, providing valuable insights into their characteristics.

### DropSens measurements on carbon electrode

**Fig. 3:**
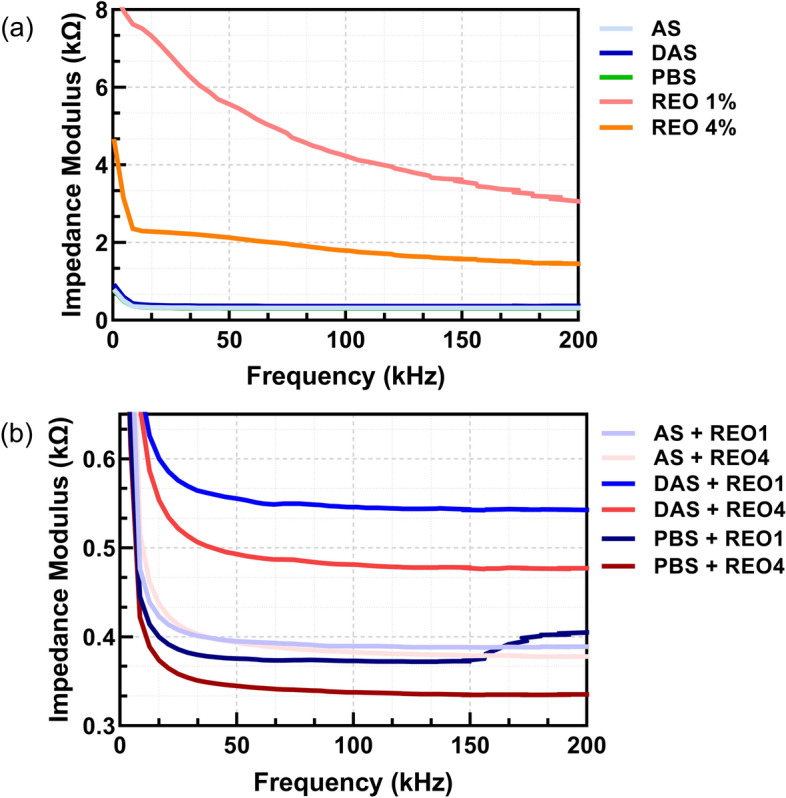
Graphs depicting impedance values in Ohms across the frequency range between 500 and 200,000 Hz for the empty electrode: **a** 5 liquids; **b** their mixtures with REO1 and REO4 carbon screen-printed electrodes, second set

The mean impedance values for the empty electrode and the various liquid samples (PBS, AS, DAS, REO1, REO4) and their mixtures (AS + REO1, AS + REO4, DAS + REO1, DAS + REO4, PBS + REO1, PBS + REO4) were analyzed across three frequency ranges: low (500 to 20,000 Hz), middle (20,000 to 100,000 Hz), and high (100,000 to 200,000 Hz). The empty electrode showed the highest mean impedance values across all frequency ranges, reflecting its baseline properties without any liquid medium. Among the liquids, REO1 and REO4 exhibited higher mean impedance values compared to PBS, AS, and DAS, with REO1 consistently showing the highest impedance, indicating a more resistive behavior likely due to the essential oil's properties, depicted in Fig. 3a. The mixtures generally showed intermediate impedance values, suggesting interactions between the essential oils and the other liquids that modulate their overall electrical properties; Fig. 3b. Statistical analysis using pairwise *t*-tests revealed several significant differences in impedance values between the samples across the frequency ranges. For instance, the empty electrode displayed significant differences (*p* < 0.001) compared to all other samples in the middle and high-frequency ranges, highlighting its distinct electrical behavior. Significant differences were also observed between pure essential oils (REO1 and REO4) and their mixtures with other liquids, particularly in the middle and high-frequency ranges. For example, mixtures like DAS + REO1 and PBS + REO4 showed significant differences when compared to their individual components, indicating unique impedance characteristics when combined. These findings underscore the importance of frequency-specific analysis in understanding the electrical properties and interactions of various liquids and their mixtures.

### SmartMF on interdigitated electrode

**Fig. 4:**
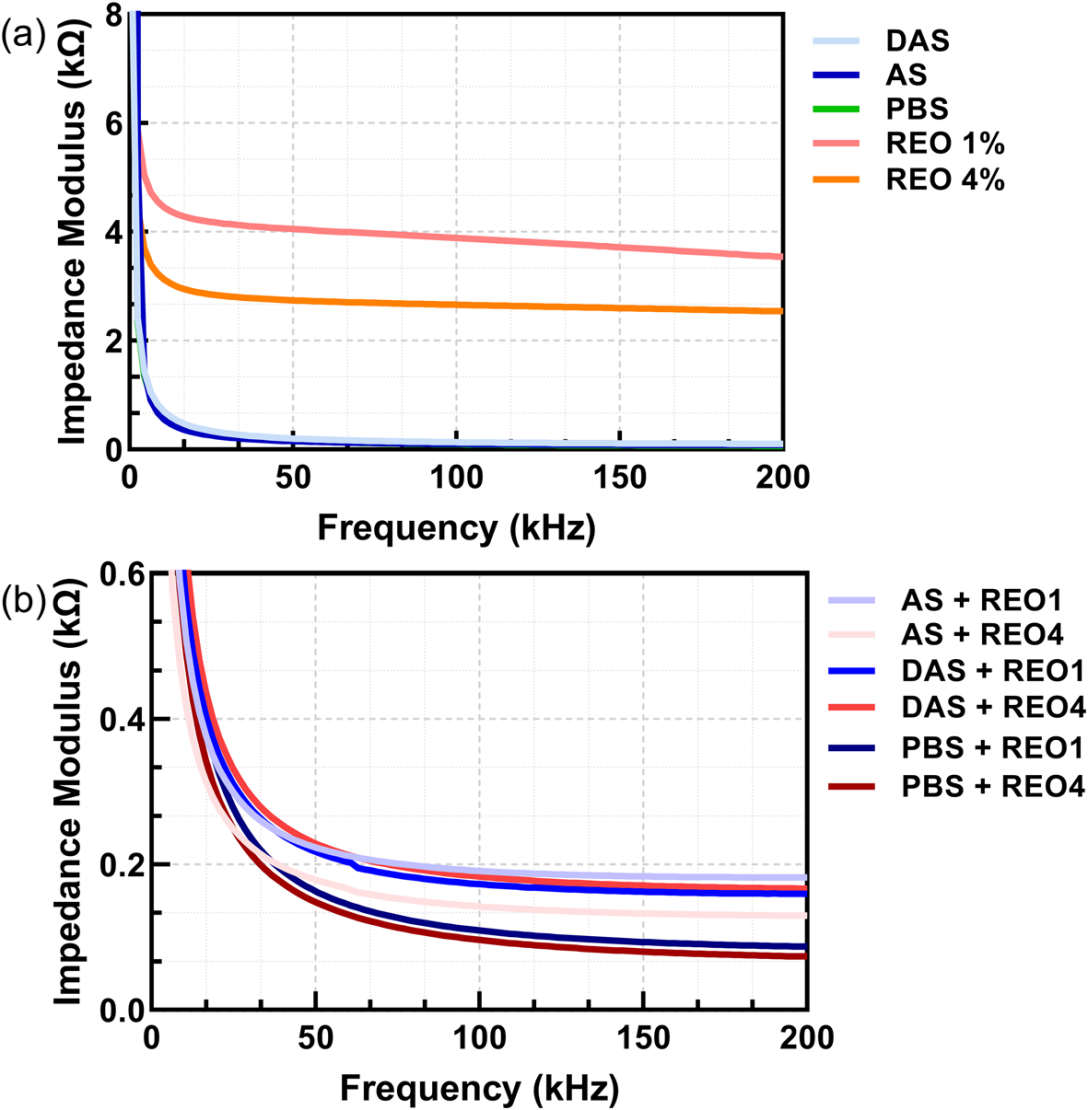
Impedance modulus measured on interdigitated electrodes: **a** with different liquids and essential oils; **b** with mixtures of these liquids and essential oils

The measurements on interdigitated electrodes, the same ones integrated into the microfluidic disk, have shown similar results in terms of trend and value with other types of electrodes presented in this paper. More precisely, a prevailing trend in all the measurements is that a higher concentration of essential oil in an emulsion leads to lower values of impedance modulus. This may result from aggregation and phase separation of the essential oils with increased concentrations, leading to a change in dielectric properties and decreased impedance overall. Furthermore, the mixtures shown in Fig. 4b show overall lower impedance values for mixtures with 4% wt. of essential oils than those with 1% wt. essential oil. Even though lower values can be seen, all impedance values are quite close to each other, and the differences are minimal, in the range of around 200 Ω.

### Comparison of the CIME electrode on DropSens and SmartMF

The Pearson correlation between the two instruments' summary statistics (mean, median, standard deviation) is 0.963. This high correlation suggests a strong positive relationship between the impedance measurements taken by the SmartMF and DropSens instruments. The summary statistics (mean, median, standard deviation) across all conditions are closely aligned between the two instruments, indicating that the measurements are generally consistent despite being taken on different devices. The strong correlation value reinforces the reliability of using either instrument for impedance measurements, as they produce similar results.

The correlation between the summary statistics (mean, median, and standard deviation) of impedance measurements taken from the CIME electrode using two different instruments, SmartMF and DropSens, is depicted in Figure S3. Each point represents a specific condition (e.g., AS, DAS, PBS, etc.), with its corresponding summary statistics plotted for both instruments. As the trend line indicates, the strong positive correlation demonstrates a high level of agreement between the two instruments' measurements across various conditions. This high correlation (*r* = 0.963) suggests that both instruments provide consistent and reliable impedance measurements, making them suitable for comparative studies in this context.

### Correlation of all electrodes

The correlation analysis between the three electrodes—CIME, carbon, and interdigitated—revealed consistently strong correlations across all conditions, including the empty electrode, individual liquids (AS, DAS, PBS, REO1, REO4), and their mixtures. For the empty electrode, the correlation coefficients were exceptionally high, with values of 0.9997 between the CIME and carbon electrodes, 0.9997 between the carbon and interdigitated electrodes, and 0.9999 between the CIME and interdigitated electrodes. These results indicate a nearly perfect agreement in the impedance measurements obtained from different electrode types, confirming the reliability of the measurement setup and the uniformity of the electrode performance in the absence of a conductive medium. Similarly, high correlations were observed when analyzing the impedance of various liquids and mixtures. For instance, the impedance of artificial saliva (AS) across the three electrodes showed a correlation of 0.950 between the CIME and carbon electrodes, 0.999 between the carbon and interdigitated electrodes, and 0.954 between the CIME and interdigitated electrodes. This trend persisted across all other liquids and mixtures, with correlation values consistently above 0.95, indicating that despite minor variations, the impedance trends remained consistent across different electrode types. These strong correlations across all conditions suggest that the three electrodes provide reliable and comparable measurements, ensuring the robustness of the experimental results across different test scenarios.

The correlation values between the impedance measurements taken from three different electrodes (CIME, carbon, and interdigitated) across various conditions, including the empty electrode, individual liquids (artificial saliva (AS), deionized artificial saliva (DAS), phosphate buffered saline (PBS), and rosemary essential oil at 1% (REO1) and 4% (REO4) concentrations) and their mixtures are shown in Figure S4. The *x*-axis represents the compared electrode pairs (CIME vs. carbon, CIME vs. interdigitated, and carbon vs. interdigitated). At the same time, the y-axis lists the different conditions under which the measurements were taken. The z-axis, represented by the height of the bars, indicates the strength of the correlation between the electrodes for each condition, with values close to 1 indicating a powerful correlation.

As can be seen from Figs. 1, 2, 3, and 4, the choice of electrode material and geometry was crucial in determining the sensitivity and accuracy of impedance measurements. The CIME electrodes, which are gold-based, exhibited high conductivity and stability in the tested solutions, making them suitable for precise electrochemical impedance spectroscopy (EIS) measurements. On the other hand, the interdigitated electrodes integrated within the microfluidic disk enabled fine control over small fluid volumes but showed lower sensitivity than the CIME electrodes. While less sensitive, the carbon electrodes provided a cost-effective and reliable option for testing large batches of samples.

### Application of electrodes on microfluidic CD—SmartMF on-disk measurements

**Fig. 5:**
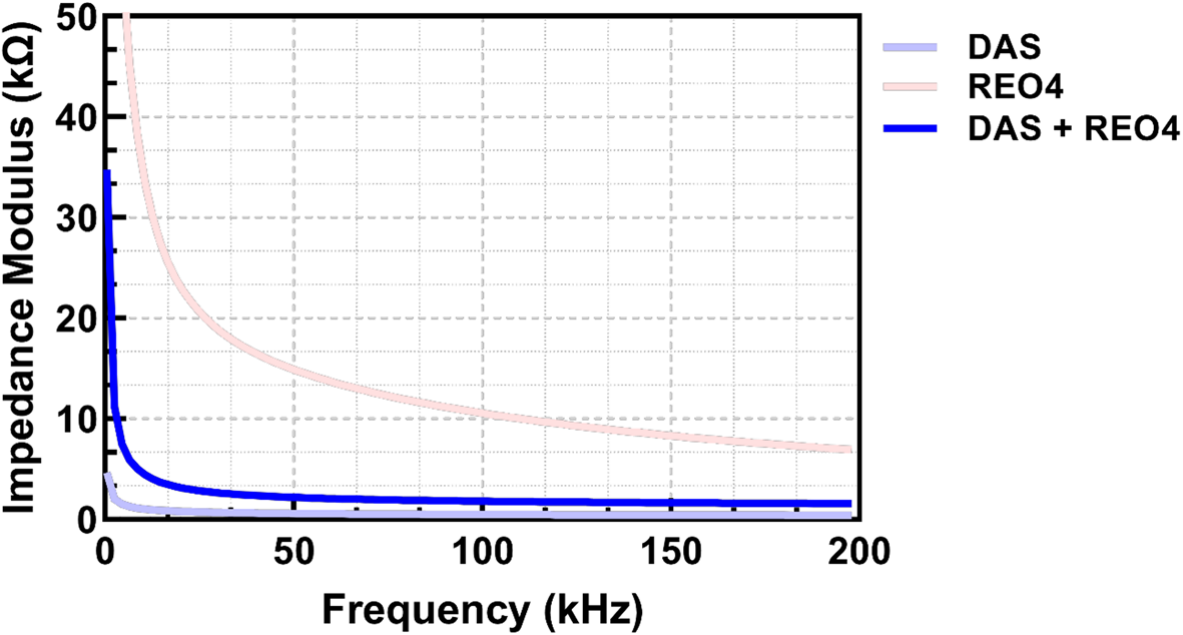
Comparison of impedance values in mixtures

The impedance analysis within the microfluidic CD across three frequency ranges for DAS, REO4, and their mixture DAS REO4 reveals distinct electrical behaviors individually and in combination, as shown in Fig. 5 and detailed in Table 3. DAS exhibits a mean impedance of 1,157.13 Ω in the low-frequency range, with a standard deviation suggesting moderate variability. REO4, in contrast, shows a significantly higher mean impedance of 39,645.27 Ω, with a very high standard deviation indicating substantial measurement inconsistency that could arise from sample heterogeneity or interactions with the measurement setup. The mixture of DAS and REO4 presents a mean impedance of 5,382.92 Ω, which is notably lower than that of REO4 alone and higher than DAS, suggesting a mitigating effect of DAS on the impedance properties of REO4, likely due to a synergistic interaction or dilution effect.

**Table 3:** Summary of impedance measurements

	Low (500 Hz–20 k Hz)	Mid (20–100 kHz)	High (100–200 kHz)
DAS mean (Ω)	1157.127	575.261	436.448
DAS SD (Ω)	389.516	81.212	19.651
REO4 mean (Ω)	39,645.273	14,539.843	8459.023
REO4 SD (Ω)	17,198.499	3274.599	1046.377
DAS REO4 mean (Ω)	5382.921	2179.514	1662.602
DAS REO4 SD (Ω)	2566.047	345.565	68.061

As the frequency increases to the mid-range, all substances show a decrease in impedance values, reflecting more stable and consistent measurements. The mixture, DAS REO4, also shows enhanced electrical stability with a mean impedance of 2179.51 Ω and a further narrowed standard deviation of 345.56 Ω.

In the high-frequency range, the trend of decreasing impedance continues. The substantial decrease in standard deviation across the frequency ranges for all substances suggests improved measurement consistency at higher frequencies. DAS consistently shows the lowest impedance values, indicating it is less resistive than REO4. The mixture of DAS and REO4 shows intermediate values, indicating interaction effects that moderate the impedance characteristics of REO4.

The findings of on-disk measurements of impedance moduli in the 500 Hz–200 kHz frequency range are displayed in Fig. 5. The highest impedance value is for REO 4% wt, which coincides with previous findings on out-of-disk samples. Moreover, pure dyed artificial saliva has a lower impedance modulus, and when mixing the two, the impedance value over the whole frequency span is between the two characteristics mentioned above.

**Fig. 6:**
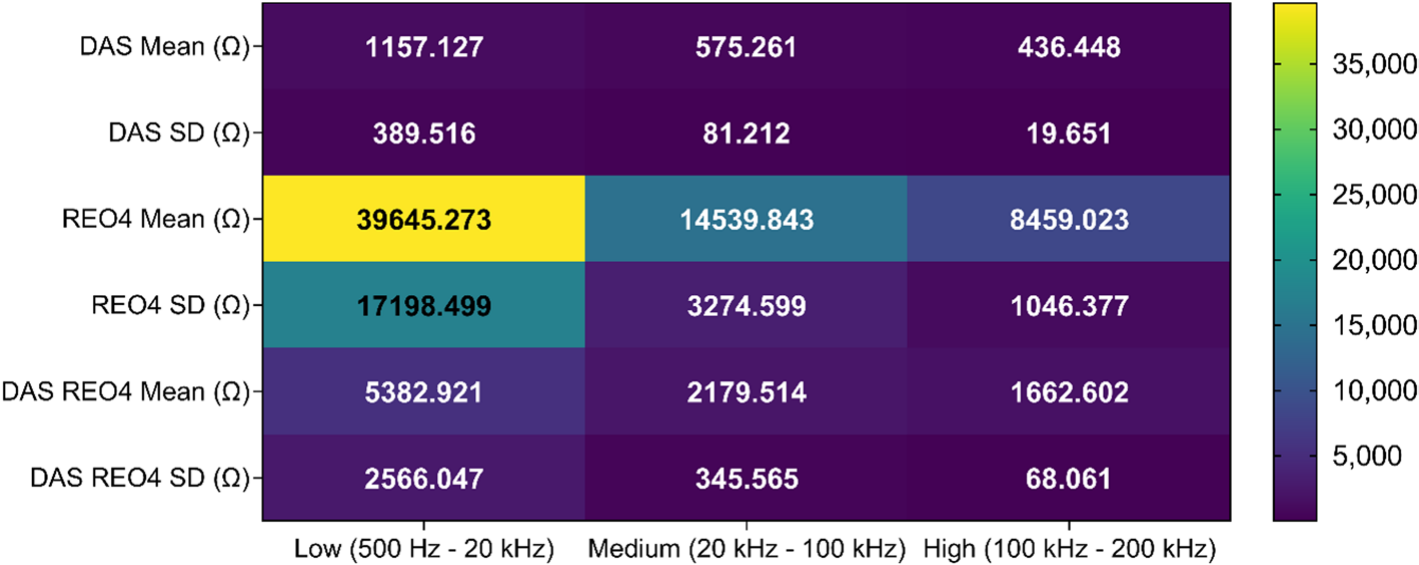
Heatmap of impedance descriptive statistics for DAS, REO4, and DAS REO4

The statistical analysis across low, middle, and high-frequency ranges shows extremely low *p*-values, indicating highly significant differences in impedance between the liquids DAS, REO4, and DAS REO4; Fig. 6. For all comparisons and frequency bands, the *p*-values are well below the common significance threshold (e.g., 0.05), suggesting that the observed differences are unlikely to be due to random chance. In the middle and high-frequency ranges, the *p*-values are notably minuscule, reaching values close to zero, especially when comparing DAS vs. DAS REO4 at high frequencies.

### Comparison of out-of-disk and on-disk measurements using the same interdigitated electrodes

**Fig. 7:**
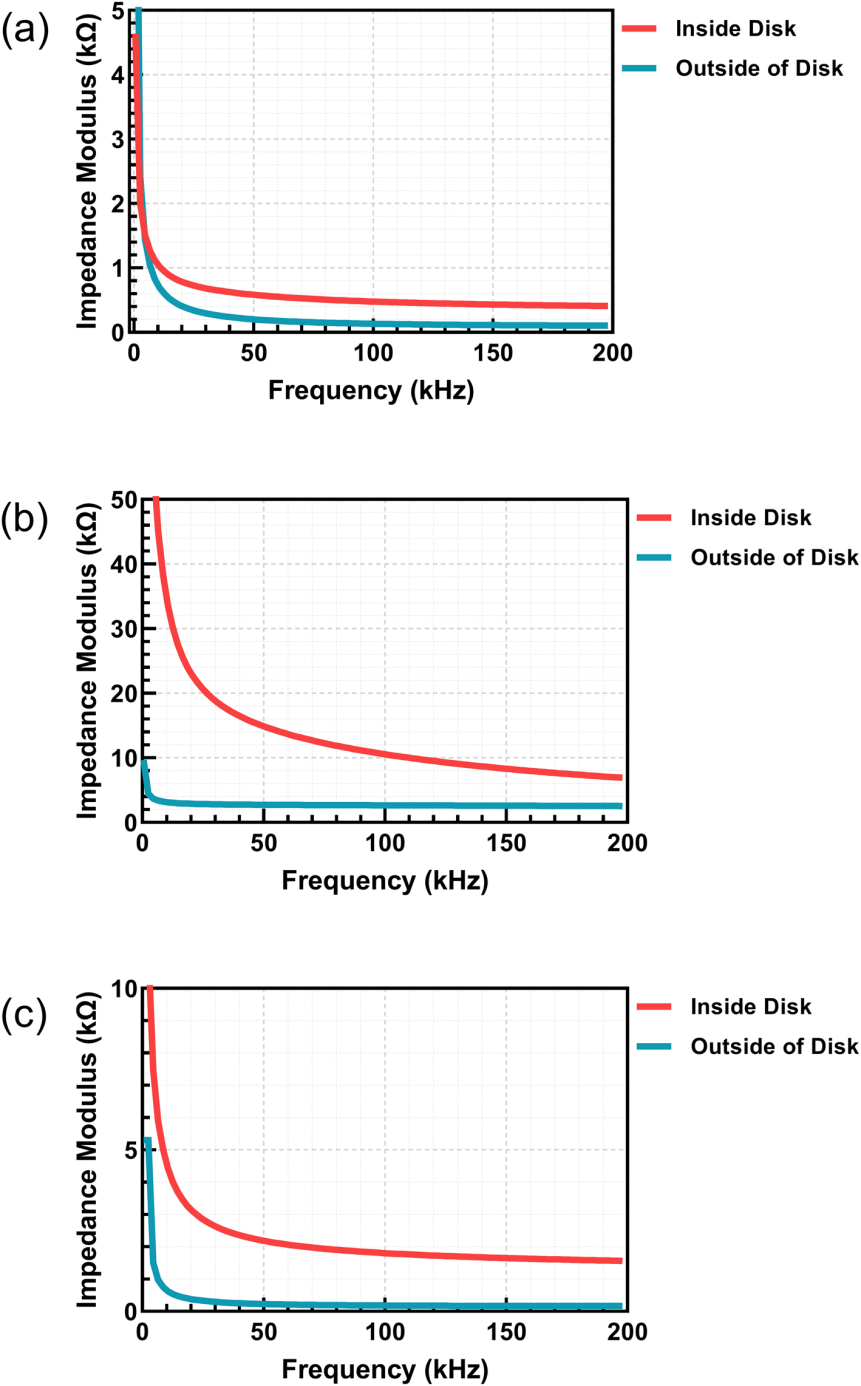
Comparison of inside of disk and outside of disk impedance values for **a** DAS; **b** REO4; **c** DAS REO4

Figure 7 compares the impedance moduli of different mixtures in and out of the disk. Across all three liquids, significant differences can be seen, as the impedance modulus is higher inside the disk compared to out-of-disk measurements. This can be attributed to resistance in conductive lines inside the microfluidic disk, which connect the electrodes to the ports of the Smart MF system, which are absent from the out-of-disk measurements.

## Discussion

This study explored the potential of rosemary essential oil (REO) in dental applications, mainly focusing on its interactions with artificial saliva (AS) and the resulting electrical impedance characteristics. By leveraging microfluidic devices and electrochemical impedance spectroscopy (EIS), we have detailed the unique electrical properties of REO and its mixtures, revealing significant implications for oral health care. The findings underscore the broader impact of integrating essential oils into dental hygiene practices, presenting a promising alternative for combating oral pathogens and enhancing overall dental health. The bioactivity of rosemary essential oil (REO) is critical in its potential application for oral health. REO's antibacterial properties, particularly against key oral pathogens such as *Streptococcus mutans* and *Escherichia coli*, are attributed to its phenolic compounds like carnosol and rosmarinic acid. These compounds disrupt bacterial cell walls, inhibiting growth and reducing plaque formation, thus promoting oral hygiene [30, 31].

The EIS results revealed substantial variations in impedance across different samples and frequency ranges, highlighting the distinct electrical properties of REO. These findings are consistent with previous studies that emphasized the unique behavior of essential oils when subjected to electrical fields. For instance, our results showed higher impedance values for REO mixtures than pure AS, which aligns with similar investigations describing that impedance variations in essential oils are influenced by sample types and frequency ranges [32]. Essential oils like tea tree, clary sage, and cinnamon bark oil show increased electrical resistance, decreased capacitance, and higher impedance with rising concentrations, allowing for rapid oil type and quality determination. Industrial lemon oil exhibits unique dielectric behavior at low frequencies, with measured permittivity and conductivity values aiding in understanding its properties [33]. Additionally, the composition of base oils like SN-100, SN-150, SN-500, and SN-650 impacts impedance changes across a wide frequency range, with different oils showing distinct impedance patterns and behaviors [34]. Furthermore, studies on water-in-crude oil emulsions reveal that impedance modulus and phase angle increase with water content, showcasing a capacitor-like behavior influenced by frequency and water concentration [35]. These findings highlight the diverse impedance responses of essential oils and related substances under varying conditions and frequencies.

The results indicated that the CIME electrodes offered the highest sensitivity, particularly at lower frequencies, due to their superior conductivity. While beneficial for their integration into the microfluidic system, the interdigitated electrodes demonstrated slightly lower accuracy, especially at higher REO concentrations. This variation can be attributed to differences in surface area and material properties between the electrodes. Future studies could explore alternative materials or geometries to enhance measurement accuracy further.

The observed impedance characteristics, particularly the higher impedance at lower concentrations, can be attributed to the formation of micelles or other structures that enhance resistance. This behavior is particularly significant in the context of oral care, as the formation of micelles could improve REO's stability and antimicrobial activity, particularly against oral pathogens like *Streptococcus mutans*. Future research should further explore how micelle size and composition affect the electrical properties of essential oil solutions. Essential oils demonstrate non-linear impedance behavior due to their complex molecular interactions. Moreover, the impedance reduction in mixtures with PBS and AS suggests a dilution effect, which reduces the overall resistance of the solution. Diluting essential oils with aqueous solutions decreases their impedance, enhancing electrical conductivity. The impedance reduction of essential oils when diluted with aqueous solutions can be attributed to the changes in electrical parameters, as demonstrated in a study utilizing knitted silver threads for detection [36]. The impedance of the solutions, measured through electrochemical impedance spectroscopy, showed an increase in electrical resistance and impedance with higher essential oil concentrations, indicating a decrease in capacitance [37]. This phenomenon aligns with the concept of electrical impedance changes in systems involving dilute aqueous electrolyte solutions, where the frequency-dependent conductance and susceptance components are analyzed to understand the electrochemical reduction mechanisms, such as the reduction of water molecules at various electrodes [32]. Therefore, the impedance reduction observed when diluting essential oils with aqueous solutions can be linked to alterations in the electrical properties of the solution due to the presence and concentration of the oils.

In our research on the electrical properties of essential oils, a particularly intriguing observation emerged: a 1% solution of essential oil exhibits greater impedance than a 4% solution. This counterintuitive finding can be elucidated by examining several factors related to the essential oil's attributes and its interactions with the solvent and electrodes. Essential oils can affect the solution's conductivity in a non-linear manner. Essential oils might form micelles or other complex structures that elevate the resistance at lower concentrations. As the concentration increases, these structures could disintegrate, resulting in a reduction in impedance. This non-linear behavior suggests that the essential oil’s molecular arrangement is crucial in determining the solution's overall electrical properties. The dielectric properties of essential oils significantly influence the impedance. The essential oil molecules may disperse more uniformly throughout the solvent at a lower concentration, leading to a higher dielectric constant and, consequently, higher impedance. In contrast, the essential oil may undergo phase separation or aggregation at higher concentrations, altering the dielectric environment and reducing impedance. This shift in the dielectric properties highlights the complex interactions between the oil molecules and the solvent.

Interactions between essential oils and ions in the solvent also contribute to the observed impedance behavior. Essential oils might hinder ion mobility at lower concentrations, increasing impedance. However, there might be sufficient essential oil at higher concentrations to form pathways or channels that facilitate ion movement, reducing impedance. These ionic interactions indicate that the essential oil concentration critically influences ion transport mechanisms within the solution.

Essential oils can alter the viscosity of the solution, impacting ion mobility. At a 1% concentration, the increase in viscosity might impede ion movement, leading to higher impedance. Conversely, at a 4% concentration, if the essential oil induces micelle formation or other structural changes that decrease the solution's viscosity, ion movement could become less restricted, resulting in lower impedance. This relationship between viscosity and impedance underscores the essential oil's role in modulating the physical properties of the solution.

At lower concentrations of rosemary essential oil (REO), the formation of micelles increases impedance, likely due to the aggregation of REO molecules, which alters the dielectric properties of the solution. As the concentration of REO increases, larger micelles are formed, encapsulating more ions, which reduces ion mobility and subsequently lowers conductivity. This behavior may also affect the local ionic environment by reducing the free ions available for conduction. However, larger micelles may carry a higher charge density, potentially enhancing conductivity under certain conditions. Furthermore, the micelle formation stabilizes REO molecules, allowing for a more controlled release of active compounds. This stabilization could improve the antibacterial efficacy of REO, particularly against common oral pathogens like *Streptococcus mutans*. These findings suggest that micelle formation not only influences the electrical properties of REO–AS mixtures, but also plays a crucial role in enhancing the antimicrobial potential of REO in oral care applications [38–41].

The interaction of essential oils with the electrode interface can influence impedance measurements. At lower concentrations, essential oils might cause more significant polarization effects at the electrode surface, thereby increasing impedance. At higher concentrations, changes like the interaction between the essential oil and the electrode surface could reduce polarization effects and thus decrease impedance. This phenomenon points to the impact of essential oil on electrochemical dynamics at the electrode interface.

The solubility and dispersion of essential oils in the solvent do not necessarily follow a linear pattern. At a 1% concentration, the essential oil might be partially solubilized to maximize impedance. At 4%, an excess of essential oil could lead to saturation effects, where additional oil fails to increase impedance proportionally and might even decrease it due to changes in the solution's microstructure. These non-linear solubility effects reveal the complex solubility behavior of essential oils in solvents.

The higher impedance observed at a 1% essential oil concentration compared to 4% can be attributed to intricate interactions among the essential oil, solvent, and electrodes. These interactions affect factors such as dielectric properties, ion mobility, viscosity, and electrode polarization in non-linear ways. Understanding these mechanisms requires further experimental scrutiny, including impedance spectrum analysis and microscopic and compositional investigations. Such studies could provide deeper insights into the specific interactions and structural changes responsible for this peculiar finding. By exploring these multifaceted interactions, our research sheds light on the complex behavior of essential oils in solutions, offering potential applications in fields ranging from bioengineering to materials science. The impedance variations observed with REO are consistent with those reported for other essential oils, such as clove and peppermint, where higher concentrations typically reduce impedance due to phase separation and reduced dielectric properties. Similar trends have been reported in dielectric gels with microphase separation studies, which also exhibit changes in electrical properties under pressure and concentration gradients [42]. The counterintuitive nature of our findings invites further inquiry and underscores the importance of comprehensive analysis in understanding the nuanced behavior of complex systems.

Using microfluidic devices to study the electrical properties and interactions of REO with AS represents a novel approach in dental research. These devices offer precise control over experimental conditions, allowing for real-time monitoring and analysis. Our findings on stability and uniform distribution of REO in saliva within microfluidic systems. Microfluidic devices play a crucial role in enhancing the accuracy and reliability of studying electrical properties and interactions, such as impedance monitoring and electrical current changes, between materials like REO and AS in dental research. These devices offer precise control over fluid flow and enable the manipulation of microscale environments, leading to improved measurements and analysis of electrical phenomena [37, 43, 44–47]. By utilizing microfluidic channels with specific electrode configurations and materials, researchers can minimize issues like joule heating, optimize medium conductivity for cell survival, and enhance sensitivity in impedance monitoring, ultimately improving the reproducibility and effectiveness of studies on REO–AS interactions in dental applications. Additionally, integrating microfluidic structures in organ-on-chip systems allows for more accurate replication of in vivo microenvironments, leading to better predictive power and long-term recordings of electrical activity in dental research settings.

Microfluidic devices are particularly valuable in dental research for several reasons. Firstly, they enable the integration of complex systems in a miniaturized state, allowing for the manipulation of fluids at a cellular scale and the control of flow rates. This level of precision is beneficial in the realm of pharmaceutical research, aiding in the development of drugs, the optimization of dosages, and the creation of controlled release systems. These capabilities provide a dynamic environment for conducting experiments and monitoring analytes.

Secondly, microfluidic devices are ideal for point-of-care diagnostic systems, particularly using saliva as a diagnostic fluid. Saliva testing is easy and accessible, and microfluidics-based methods can save time and cost compared to traditional methods. Saliva-based diagnostics can provide a non-invasive, cost-effective method for monitoring oral and systemic health. The ability of microfluidic systems to accurately analyze saliva samples for various biomarkers enhances their potential for early detection and management of oral diseases. These devices offer rapid, accurate, cost-effective multi-disease detection capabilities, utilizing optical techniques like fluorescence, absorbance, and surface plasmon resonance [48]. They enable the simultaneous analysis of different types of saliva and mouthwashes, aiding in the early diagnosis and monitoring of oral diseases through electrochemical impedance analysis [27]. Additionally, microfluidic platforms integrate analyte sampling, sensing, and signaling into a single-step procedure, enhancing the detection of infectious and non-infectious diseases through nucleic acid testing [49]. Furthermore, hand-held microfluidic devices with integrated filtration membranes allow for the electrochemical monitoring of multiple salivary biomarkers, demonstrating efficacy in saliva-based point-of-care analysis for systemic health monitoring [50]. The prospects of microfluidic devices in point-of-care diagnostics involve leveraging saliva as a non-invasive biofluid for detecting biomarkers, reflecting those in blood, to enhance further the sensitivity and selectivity of disease-specific biomarker detection [51].

Thirdly, microfluidic devices can study dental biofilm formation, offering control over multiple microenvironmental factors and allowing for quantitative analysis of bacteria growth and biofilm development. This is important for understanding the conditions that promote colonization and biofilm formation, which are critical for addressing tooth decay and related health problems. The work of Thiha et al. [27] supports this application, demonstrating the use of microfluidic CDs to investigate electrochemical property changes between artificial and real salivary samples mixed with mouthwashes using electrical impedance analysis.

The integration of REO into oral care products could revolutionize dental hygiene practices. Its antimicrobial, anti-inflammatory, and antioxidant properties make it an ideal candidate for preventing and treating oral diseases. The pleasant aroma of rosemary oil enhances user acceptance, making it a desirable ingredient in mouthwashes, toothpaste, and other dental care products. Our findings suggest that REO could be particularly effective in combating oral pathogens like *Streptococcus mutans* and *Candida albicans*, which are major contributors to dental caries and oral infections. The reduced impedance observed in mixtures of REO with PBS and AS indicates that REO can be effectively incorporated into aqueous solutions, maintaining its therapeutic properties while enhancing its electrical conductivity. This is crucial for developing oral care products that are both effective and easy to use.

Furthermore, the ability of REO to modulate the immune response and reduce inflammation suggests its potential in treating periodontal diseases [23, 52]. Periodontitis is a common chronic inflammatory condition that affects the gums and supporting structures of the teeth, leading to tooth loss if untreated. The anti-inflammatory effects of REO could help mitigate the progression of periodontitis, improving oral health outcomes for individuals suffering from this condition. The antioxidant properties of REO also offer protection against oxidative stress, which is linked to various oral health issues, including oral cancer [53, 54]. The ability of REO to scavenge reactive oxygen species and protect against oxidative damage suggests its potential to prevent oral cancer and other oxidative stress-related conditions.

Future research should focus on long-term clinical trials to evaluate the safety and efficacy of REO in oral care. While our study provides valuable insights into the potential of REO, in vitro experiments cannot fully replicate the complex environment of the oral cavity. Clinical trials involving human participants are necessary to confirm REO's therapeutic benefits and determine the optimal concentrations for use in oral care products.

Rosemary essential oil (REO) has the potential to be combined with other essential oils to enhance its efficacy in oral health applications. For instance, combining REO with clove oil, known for its strong antibacterial properties, could broaden the spectrum of antimicrobial activity against oral pathogens, such as *Streptococcus mutans*. In addition, blending REO with anti-inflammatory oils like chamomile or lavender could further reduce gum inflammation, alleviating symptoms of periodontal disease. Enhanced antioxidant protection may also be achieved by combining REO with oils like cinnamon or thyme, providing a comprehensive defense against oxidative stress in the oral cavity. These combinations offer promising opportunities for creating multifunctional dental care products aimed at both prevention and treatment of oral diseases [15, 55–59].

The potential of microfluidic devices for personalized dental care should also be further investigated. These devices offer the ability to conduct real-time monitoring and analysis of saliva samples, providing valuable information about an individual's oral health status. By leveraging microfluidic technology, it may be possible to develop personalized oral care products tailored to the specific needs of each individual, enhancing the effectiveness of treatment and improving oral health outcomes.

While our study provides valuable insights into the potential of REO for dental applications, it has certain limitations. The in vitro nature of the experiments may not fully replicate the complex environment of the oral cavity. Moreover, the concentration-dependent effects observed in impedance measurements warrant further investigation to determine the optimal concentrations for therapeutic use. Future studies should include in vivo experiments to validate our findings and explore the long-term effects of REO on oral health.

One limitation we encountered was maintaining consistent flow rates of the viscous REO–AS mixtures through the microfluidic channels. This variability could have influenced the impedance measurements, particularly at lower frequencies. Additionally, the small volumes used in microfluidic systems may only partially replicate the complexity of the oral environment, potentially affecting the generalizability of the results to real-world applications. However, these limitations are relevant to the overall findings, and future work should focus on optimizing the microfluidic system for more reliable measurements. There were also reports that oil-based solutions flow spontaneously into microchannels due to wettability issues [60, 61].

Another limitation is the variability in the composition of essential oils, which can affect their therapeutic properties. The chemical composition of REO can vary depending on factors such as the plant's geographical origin, the method of extraction, and storage conditions. Standardizing the composition of REO and other essential oils used in research and commercial products is vital to ensure consistent results and efficacy.

## Conclusion

In conclusion, our study demonstrates the potential of rosemary essential oil in dental applications. Its electrical properties and antimicrobial, anti-inflammatory, and antioxidant effects position it as a valuable candidate for enhancing oral health. The use of microfluidic devices further underscores the innovative approaches in dental research, offering precise and reliable measurements. By integrating REO into dental care regimens, we can harness its therapeutic properties to combat oral pathogens, reduce inflammation, and protect against oxidative stress, ultimately enhancing oral health. To validate the efficacy of rosemary essential oil (REO) in oral care products, randomized controlled trials (RCTs) will be necessary. These trials should compare the effectiveness of REO-based products against conventional oral care formulations in preventing dental issues such as plaque formation and gingivitis. The primary outcomes should include reductions in plaque index, gingival index, and microbial load, focusing on key pathogens like *Streptococcus mutans*. Additionally, long-term studies will be needed to assess the safety, user acceptability, and potential adverse effects of prolonged use of REO-based products. These clinical trials will provide the necessary evidence to support the incorporation of REO into mainstream oral care products.

## Material and methods

### Sample preparation

Artificial saliva (AS) was composed of carboxymethyl cellulose following the recipe provided by the Pharmacy Institution Belgrade, which is registered under the Republic of Serbia’s master preparations. This ensured consistency and reliability in the composition of the AS throughout the study. Dyed artificial saliva (DAS) was prepared as 0.1% Cobalt Blue dye (a commercially available coloring agent) to artificial saliva, providing a visually distinct medium for experimental analysis.

Rosemary essential oil (REO) was sourced from the esteemed Institute Josif Pančić, Serbia, known for its quality and purity. REO-based nanoemulsions were formulated using adequate amounts of essential oil, Polysorbate 20, and water by the spontaneous, low-energy emulsification method (described by Pavlović et al., 2024—unpublished data). Two concentrations of REO were utilized: 1% (REO1) and 4% (REO4), enabling the investigation of concentration-dependent effects on impedance characteristics.

Phosphate buffer solution (PBS), provided by AAT Bioquest, maintained at a pH of 7.4, served as a standard electrolyte medium. Its consistent composition ensured stability and reproducibility in the experimental setup.

### Liquid mixtures

To explore potential interactions and effects of mixing different liquids, various mixtures were prepared:

- 50% AS and 50% REO1 mixture
- 50% DAS and 50% REO1 mixture
- 50% AS and 50% REO4 mixture
- 50% DAS and 50% REO4 mixture
- 50% PBS and 50% REO1 mixture
- 50% PBS and 50% REO4 mixture.

### Electrical impedance spectroscopy analysis

Electrical impedance spectroscopy (EIS) was conducted on all liquid samples using DropSens Metrohm (Metrohm DropSens, Oviedo, Spain) and SmartMF, CIME, Malaysia [29], Supplementary files—Figures S1 and S2). The DropSens Metrohm is a widely used device for electrochemical analyses, valued for its precision and reliability in various research and industrial applications.

**Fig. 8:**
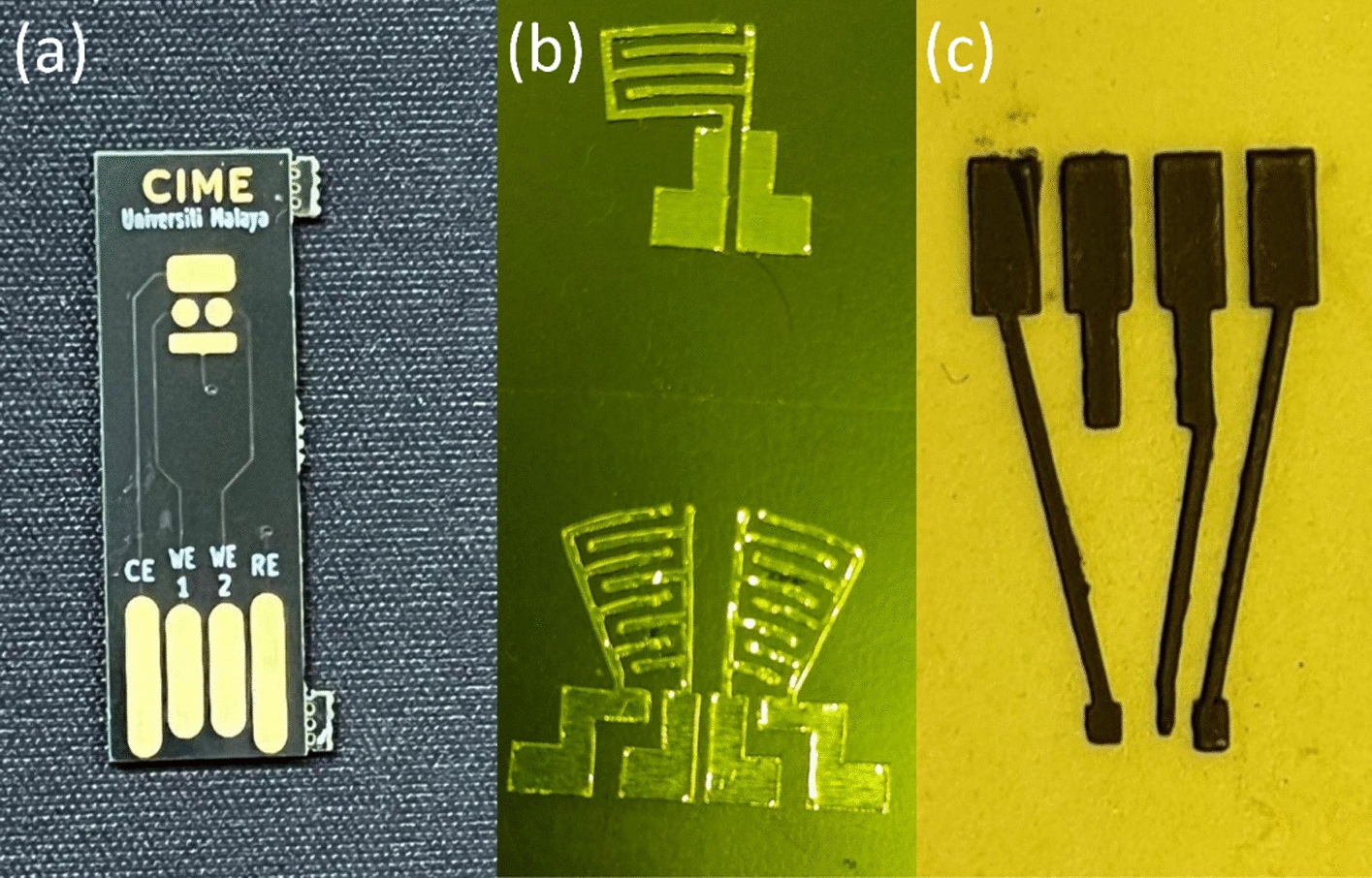
Electrodes used for the EIS measurement: **a** CIME electrode [30]; **b** interdigitated electrodes used for the on-disk EIS measurements; **c** carbon-based screen-printed electrodes

We also utilized our in-house-designed sensor chip, which was fabricated using the standard printed circuit board (PCB) technique. The sensor chip adopts the conventional three-electrode system, i.e., working, counter, and reference electrodes (WE, CE, and RE, respectively) made of gold (Au), as shown in Fig. 8a.

As mentioned, apart from the widely used DropSens, we utilized a custom-designed impedance analyzer to test its reliability, employing three different types of electrodes for comprehensive evaluation. In Fig. 8, the in-house fabricated electrochemical electrode CIME (a) is shown, and the interdigitated aluminum electrodes (b) are used inside the microfluidic disk for testing and the comparison for in and out disk measurements. Finally, in-house fabricated screen-printed carbon electrodes, as shown in Fig. 8c, have also been used.

EIS measurements were performed over a specified frequency range of 500 Hz to 200 kHz to capture the electrical response of the liquids.

### On-disk measurement

**Fig. 9:**
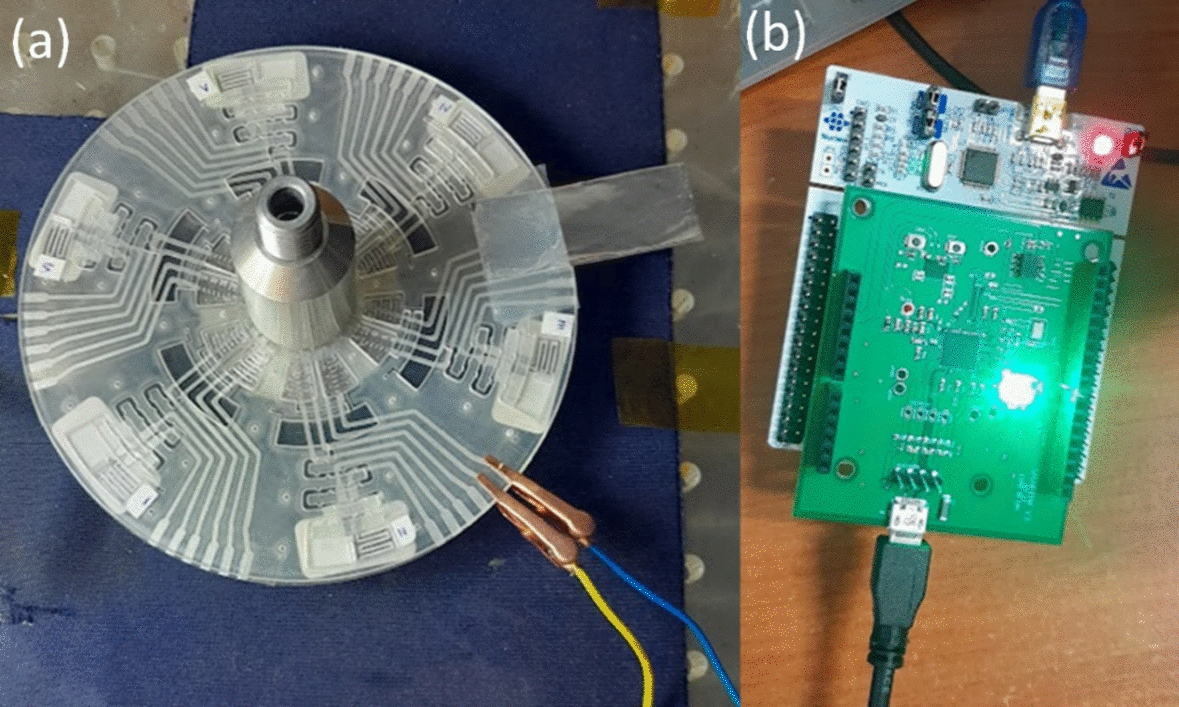
EIS measurement on-disk: **a** connections between disk and the Smart MF system; and **b** smart MF system

The fabrication of a microfluidic compact disc (CD) through the implementation of the xurographic technique is detailed in this investigation. A previously established microfluidic CD design involving polyvinyl chloride (PVC) foils shaped using xurography was used, as discussed in a prior publication [27]. This CD comprises seven PVC foil layers specified in Fig. 9, with layer compositions and functionalities consistent with the initial description [27]. Layers 1, 6, and 7 feature 80 μm PVC foils for structural support and electrode integration, while layers 2 to 5 consist of 250-μm foils forming the microfluidic channels and chambers.

The manufacturing process maintained uniformity by utilizing identical equipment and parameters, such as the Graphtec CE6000-60 plus cutter plotter and a 45° cutting blade, ensuring the consistency of CD production. The alignment and lamination of each layer followed standardized procedures to guarantee accuracy and coherence in the final stacked configuration.

In this investigation, the microfluidic CD was adapted for the evaluation of two specific liquid reagents, DAS and REO4. The CD's design aimed to enable the concurrent or repetitive analysis of these reagents within a controlled setting. In contrast to previous applications involving various saliva and mouthwash combinations, this study focused specifically on DAS and REO4 in a dual experimental arrangement. The operational stages, including LOAD, HOLD, MIX, and ANALYZE, were preserved from the original design, as depicted in Fig. 9. During the LOAD phase, DAS and REO4 were introduced into the system. Rotation of the disc facilitated the transfer of liquids to the HOLD chamber, leading to their advancement into the MIX chamber, benefitting from the serpentine channel's layout that enhances mixing efficiency. The final ANALYZE phase allowed for the precise separation and analysis of the blended solutions.

**Fig. 10:**
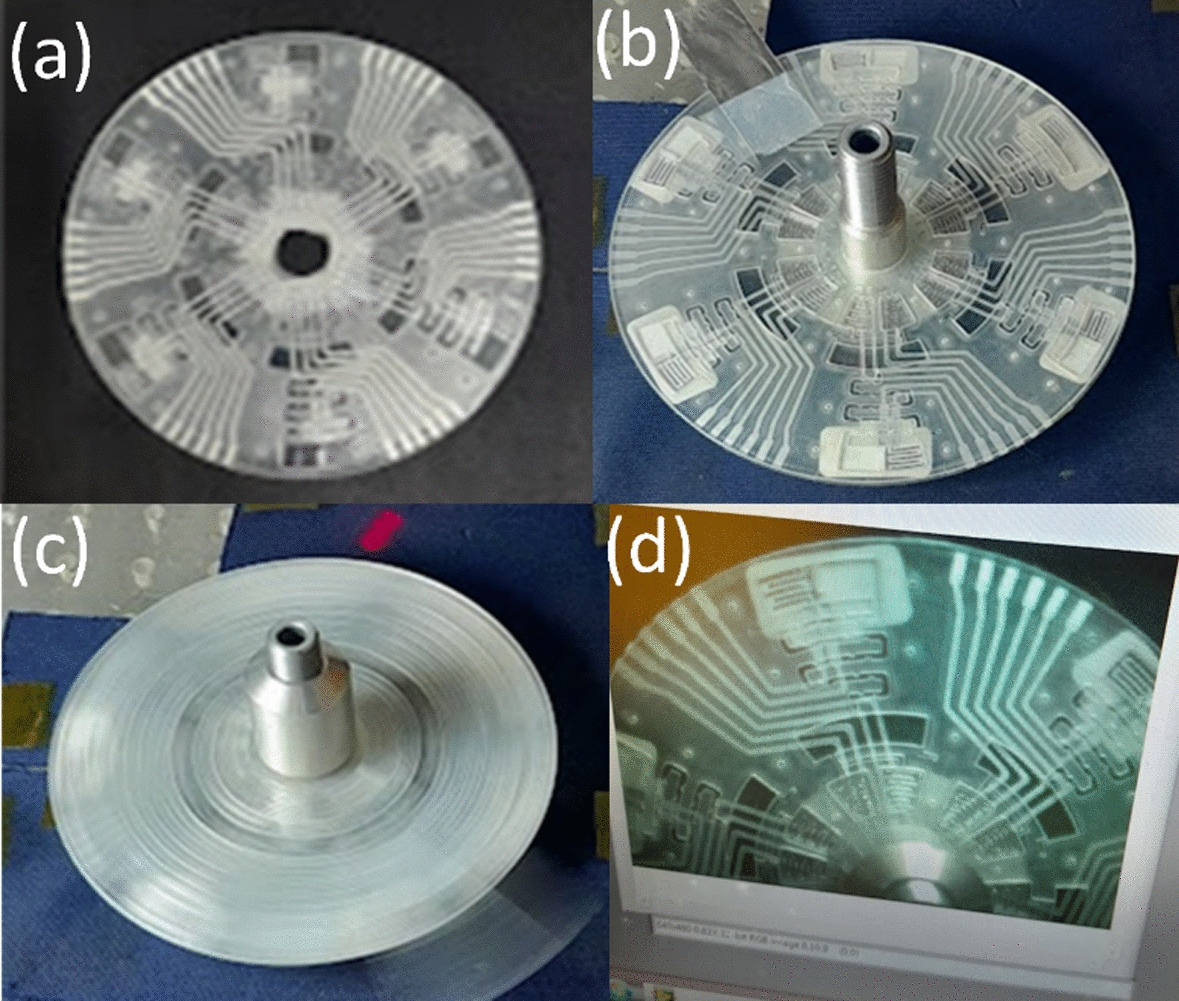
The microfluidic disk: **a** fabricated disk; **b** the disk with prepared microfluidic chambers for DAS and REO4 mixing; **c** disk spinning; **d** mixing process video acquisition

**Fig. 11:**
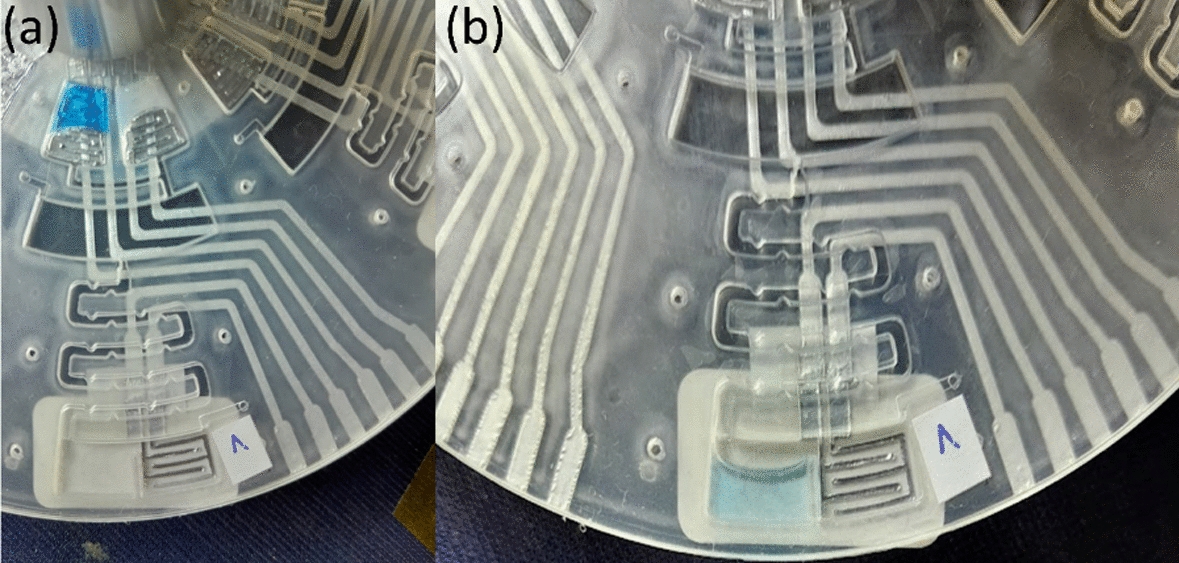
Visual objectivization of the mixing process. **a** Before spinning; **b** after spinning

The measurement approach remained consistent with the established methodology. The CD incorporates three aluminum electrodes per set, which is essential for impedance analysis carried out using the SmartMF Impedance Analyzer. This analysis evaluated the interactions between DAS and REO4, examining impedance, capacitance, and conductance at designated intervals and configurations within the CD (Fig. 10). Complementing the quantitative impedance measurements, a 18, 26comprehensive visual documentation of the ultimate mixed solution in the ANALYZE chamber provided supplementary qualitative data. The results aim to elucidate the distinct behaviors of DAS and REO4 under controlled microfluidic conditions (Fig. 11).

### Data analysis

Impedance spectra obtained from EIS measurements were analyzed to extract relevant parameters such as impedance magnitude, mixing efficacy, and correlation between electrodes and devices. Statistical methods were employed to compare the impedance characteristics of different liquid samples and mixtures. We incorporated assistance from ChatGPT. ChatGPT helped develop tailor-made visual depictions and improve the display of data, augmenting the thorough analysis carried out using GraphPad Prism [62].

## Supplementary Information

Supplementary Material 1.

## Data Availability

No datasets were generated or analyzed during the current study.

## Abbreviations

AS: Artificial saliva
DAS: Dyed artificial saliva
REO: Rosemary essential oil
EIS: Electrochemical impedance spectroscopy
PBS: Phosphate buffer solution
WE: Working electrode
CE: Counter electrode
RE: Reference electrode
PCB: Printed circuit board
CD: Compact disc
CIME: Centre for Innovation in Medical Engineering
NCDs: Noncommunicable diseases
