# Supplementary material for: Microfluidic and impedance analysis of rosemary essential oil: implications for dental health
Source: Biomed Eng Online. 2024 Nov 4;23:111. doi: 10.1186/s12938-024-01301-4 (PMC11533331; doi:10.1186/s12938-024-01301-4)
Supplement: Supplementary file 1 — Supplementary Material 1. [file 12938_2024_1301_MOESM1_ESM.docx]

Supplementary files


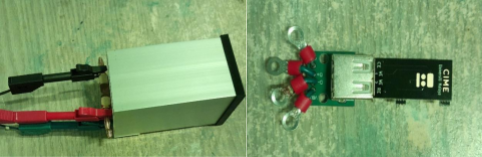


Figure S1: Dropsense EIS device setup


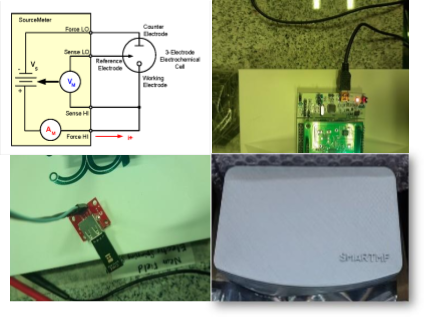


Figure S2: Smart MF EIS device setup


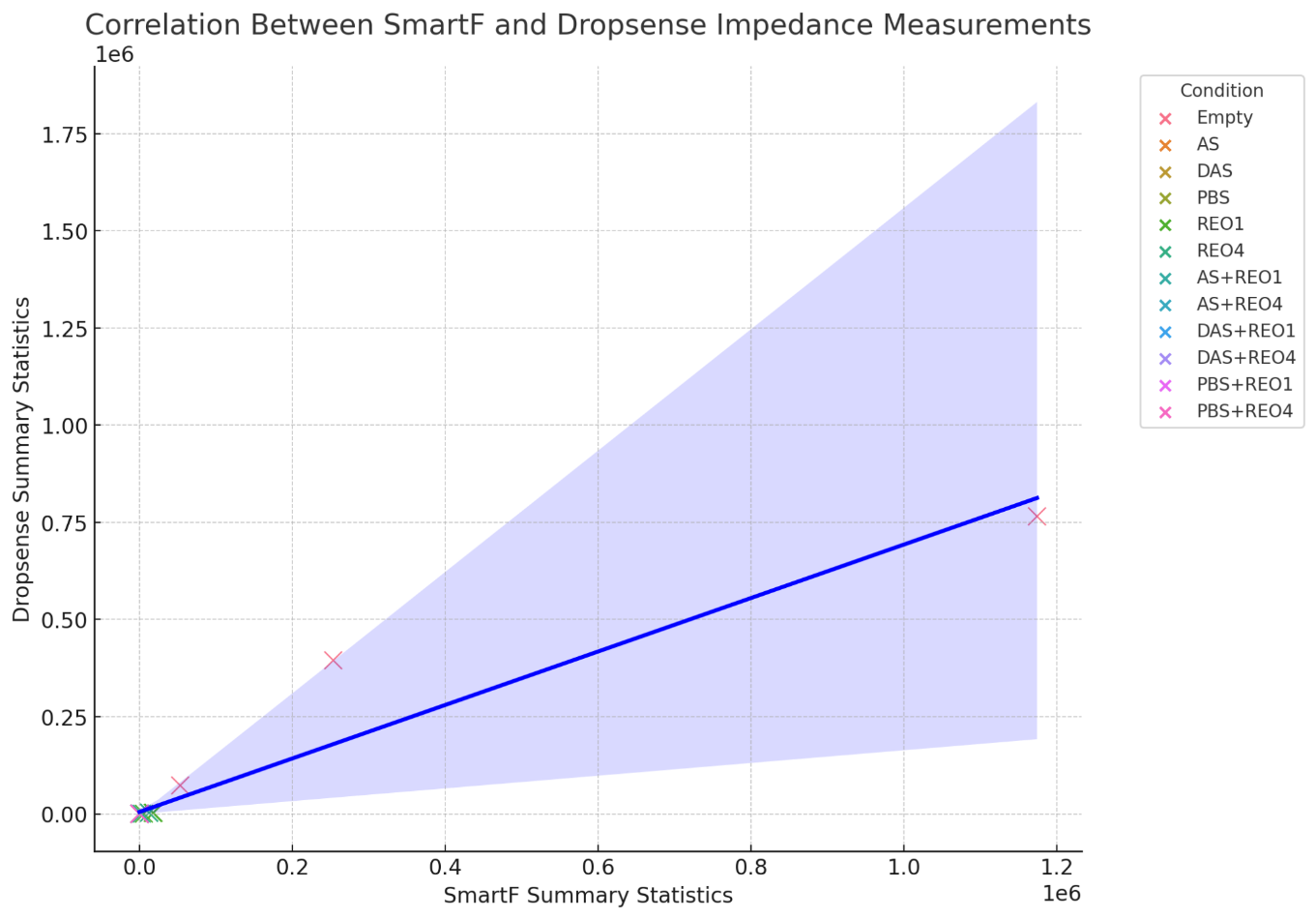


**Figure S3: Correlation Between Summary Statistics of Impedance Measurements from SmartMF and Dropsense Instruments.**

**
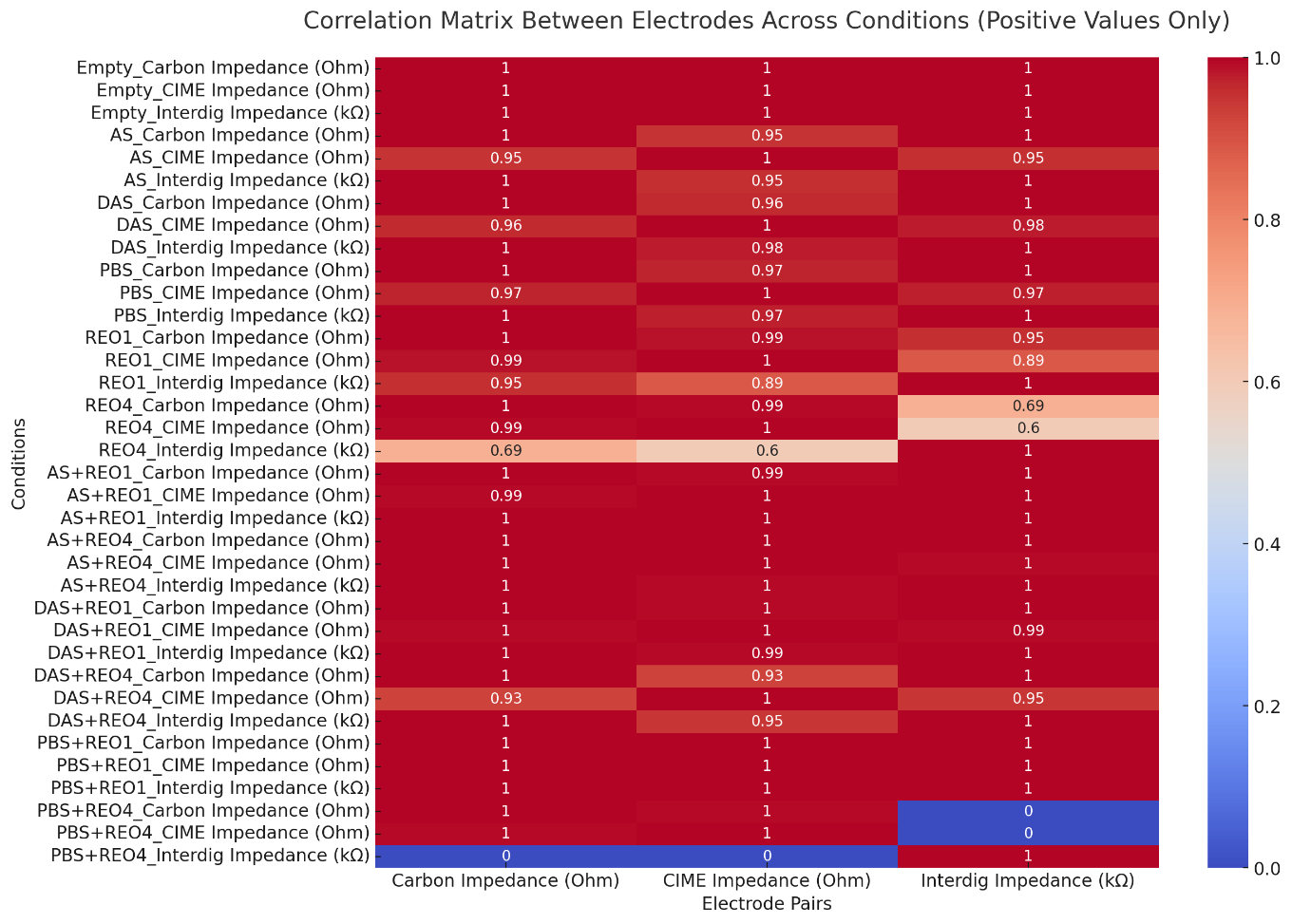
**

**Figure S4: Heatmap Visualization of Correlation Between Electrodes Across Various Conditions.**
